# Investigating the association between social interactions and personality states dynamics

**DOI:** 10.1098/rsos.170194

**Published:** 2017-09-20

**Authors:** Didem Gundogdu, Ailbhe N. Finnerty, Jacopo Staiano, Stefano Teso, Andrea Passerini, Fabio Pianesi, Bruno Lepri

**Affiliations:** 1 Fondazione Bruno Kessler, Trento, Italy; 2 Department of Information Engineering and Computer Science, University of Trento, Trento, Italy; 3 Fortia Financial Solutions, Paris, France; 4 EIT Digital, Trento, Italy; 5 Department of Clinical, Educational and Health Psychology, University College London, London, UK

**Keywords:** personality states, social interactions, ego-centric graphlets, experience-sampling method, wearable sensing, linear mixed models

## Abstract

The recent personality psychology literature has coined the name of *personality states* to refer to states having the same behavioural, affective and cognitive content (described by adjectives) as the corresponding trait, but for a shorter duration. The variability in personality states may be the reaction to specific characteristics of situations. The aim of our study is to investigate whether specific situational factors, that is, different configurations of face-to-face interactions, are predictors of variability of personality states in a work environment. The obtained results provide evidence that within-person variability in personality is associated with variation in face-to-face interactions. Interestingly, the effects differ by type and level of the personality states: *adaptation* effects for Agreeableness and Emotional Stability, whereby the personality states of an individual trigger similar states in other people interacting with them and *complementarity* effects for Openness to Experience, whereby the personality states of an individual trigger opposite states in other people interacting with them. Overall, these findings encourage further research to characterize face-to-face and social interactions in terms of their relevance to personality states.

## 1. Introduction

Although it is difficult to dispute that what people do depends both on who they are—their dispositions such as personality traits—and the situations they are involved in, psychologists have dedicated enormous amounts of effort to two competing perspectives on human behaviour and its determinants: the *person*-perspective and the *situation*-perspective [1–3]. The *person*-perspective holds that a given person will act similarly over time, and such behavioural stability makes it meaningful to describe a person in terms of general *traits*. Conversely, the *situation*-perspective claims that situations are primarily responsible for determining behaviour; hence, the focus is on the high within-individual behavioural variability over time.

Following the *person*-perspective, personality psychology has developed a view of personality as a high-level abstraction encompassing *traits*, which are sets of stable dispositions towards an action, belief and attitude formation. Personality traits differ across individuals, are relatively stable over time and can influence behaviour. Between-individual differences in behaviour, belief and attitude can therefore be captured in terms of the personality traits that are specific to each individual, providing a powerful descriptive and predictive tool that has been widely exploited by clinical and social psychology [4], educational psychology [5] and organizational studies [6,7].

The most influential example of this approach is the Five Factor model [8,9], which owes its name to the five traits it takes as constitutive of people’s personality: Extraversion, Emotional Stability, Agreeableness, Conscientiousness and Openness to Experience. Over the last 50 years the Five Factor model has become a standard in psychology, and studies using the Five Factor model have repeatedly confirmed the influence of personality traits on many aspects of individual behaviour including leadership [10], general job performance [11], sales ability [12] and teacher effectiveness [13]. Other studies have shown that subjective well-being is also related to the five factors of personality, especially Extraversion, Emotional Stability and Conscientiousness [14–16].

A distinctive feature of the *person*-perspective approach to personality is that it assumes a direct and stable relationship between traits and their associated behaviours, such that being an extravert means behaving in a way that has been characterized as being extravert (e.g. speaking loudly, being talkative). By contrast, the *situation*-perspective argues that, because the immediate situation is the primary determinant of behaviour, a given individual will act very differently on different occasions [17–20]. It can be argued that extraverts may often be silent and reflective and not talkative at all, while introverts may at times exhibit extraverted behaviours. For example, when a person who is acting extraverted at a party subsequently goes to a meeting, they may start acting introverted.

Unsurprisingly, one way of reconciling these two apparently opposing views is to maintain that both have their grain of truth to contribute: it is true that the average individual routinely expresses all the various levels of the various traits, and this within-individual variability is due to individual different sensitivities to the various situational cues. Hence, focusing on the *person-situation interaction*, rather than on the *person-situation competition*, personality psychology is moving towards a more complete understanding of why people do what they do [21–23]. For example, researchers have explored the idea of *person-situation interaction* by viewing ‘the person’ in terms of dispositions tuned by the context, rather than broad dispositions across situations [24,25], by focusing on variations of behaviour within rather than across persons [20,26–30], by theoretically integrating traditional personality traits concepts (i.e. stable cross-situationally broad dispositions) with highly contextualized psychological constructs (e.g. Fleeson and Jayawickreme’s whole trait theory [31] and other works [32,33]), and finally by investigating how people shape situations [21,34–36].

In particular, the personality psychology literature has coined the name of *personality states*, namely states having the same behavioural, affective and cognitive content (described by adjectives) as the corresponding trait, but for a shorter duration [26,27,37]. In other words, a *personality state* is a specific episode wherein a person behaves, feels and thinks as more or less introvertedly/extravertedly, more or less neurotically, etc. Personality can then be reconstructed through density distributions over personality states, which is specific to the individual, through distribution parameters such as mean, standard deviations, etc.

Previous work revealed three general findings regarding the nature of Big Five personality density distributions [26,28]. The first one is that the high amount of behavioural variability of the average individual across two weeks is almost the same as the amount of variability between individuals. This finding provides evidence of the variability in personality-related behaviours. Second, although each individual shows considerable variability, each one has a central point or tendency around which they vary. Interestingly, these central points remain stable from week to week. As pointed out in [28], the central point or tendency is consistent with a model of a trait as the causal force that determines behaviour in conjunction with varying situational forces (e.g. [38]). Finally, individuals differ not only in the central points but also in the size of their personality density distributions. These three findings together provide evidence that it is important to treat personality as having both variability in behaviour (at the moment-to-moment scale) and stability in behaviour (at the week-to-week scale) [26,27].

The variability in behaviour (i.e. switching from introverted to extraverted states from moment to moment) may be the reaction to specific characteristics of situations. Some studies have provided initial evidence that there are characteristics of situations that provoke a change in *personality states* [37,39–42]. Recently, Alshamsi *et al.* [42] have shown that a *contagion* metaphor [43], seen as the adaptation of behavioural responses to the behaviour of other people, cannot fully capture the dynamics of personality states. The authors found that social influence has two opposing effects on personality and emotional states: *adaptation* effects, whereby the personality and emotional states of an individual trigger similar personality and emotional states in other people interacting with them, and *complementarity* effects, whereby the personality and emotional states of an individual trigger opposite personality and emotional states in other people interacting with them [42,44].

Specifically, Alshamsi *et al.* [42] have identified more nuanced effects like *attraction*, *inertia* *repulsion* and *push*. Both *attraction* and *inertia* represent a tendency of individuals (egos) to adapt to the behaviours of others (alters). In *attraction* alters are in levels that are different from egos and therefore they encourage people to move to their levels; whereas, in *inertia* alters are in the same levels of egos and encourage them to stay at the same levels. Instead, *repulsion* and *push* represent the tendency of individuals (egos) to complement the behaviours of others (alters). In *repulsion* alters are in levels different from egos and therefore discourage people to move to their levels; whereas in *push* alters are in the same levels of egos and push people away from their levels.

Furthermore, these effects may exhibit different directions depending on the stable personality traits of target individuals. For example, while extrovertedly acting alters bring an introvert out of their shell, they push already extroverted persons towards introversion. However, Alshamsi *et al.* [42] assume that the personality states’ dynamics of an individual are influenced by the number of individuals in a given state they come in contact with.

The aim and the novelty of our study is to investigate whether and to what extent the different structural characteristics of the face-to-face interaction network are predictors of the dynamic personality states of a given individual (the *ego*). Based on this research question, we formulate the following hypotheses:

*Hypothesis 1*. The variability in personality-related behaviours (i.e. moving from introverted to extraverted states from moment to moment) will be associated with variation in face-to-face interactions;
*Hypothesis 2*. The different structural configurations of the face-to-face interaction network will reveal *attraction*, *inertia*, *repulsion* and *push* effects;
*Hypothesis 3*. These effects will differ by type and level of the personality states.

To this end, we represent interactions between people as *graphlets* [45], namely induced subgraphs representing specific patterns of interactions, and design regression analyses with the target of predicting dynamic transitions of participants’ self-reported personality states. Graphlets have extensively been employed to study properties of biological networks, for example, to discover invariant patterns characterizing specific properties of enzymes and small molecules [46]. Allowing to capture the local structure of interactions, graphlets represent a promising methodology to study interactions between humans. More specifically, we investigate graphlets centred on the individual under consideration (the *ego*), embedding information on the state of the other individuals they are connected with (the *alters*) and the interactions between them.

To measure the face-to-face interactions, we exploit high-resolution wearable sensors, which have previously made possible to collect enormous amount of social interaction data [47,48], alleviating the reliance on subjective self-reports based on human memory. Moreover, sensors can log data at very fine temporal granularity without interfering with peoples’ routines or consuming their time, thus making it easier to investigate short-duration phenomena, such as personality states.

## 2. Material and methods

### 2.1. Participants

A total number of 54 employees of a research centre located in northern Italy were recruited on a voluntary basis to participate in a six-week long study (only working days were considered). During introductory meetings, they were provided with detailed information about the purpose of the study; the data treatment and privacy enforcement strategies adopted; and the devices they would be using and the measurements they provide. Following Italian regulations, all participants signed an informed consent form approved by the Ethical Committee of Ca’ Foscari University of Venice.

The 54 participants belonged to five units; four units were research groups of varying size, while members of the fifth unit were part of the full-time IT support staff. The heads of the units, all the employees within three units, and the majority of the employees within the remaining two units (7 of 10 and 17 of 18, respectively) participated in the study. The ages ranged from 23 to 53 (*M*=36.88, *s*.*d*.=8.54). Forty-seven of the participants were men while only five were women; given the nature of the research institute, it was not unusual to have a smaller sample of women participating in the study. The majority of participants, 46 (84%), were of Italian nationality.

### 2.2. Procedure

During this six-week period participants used high-resolution wearable sensors, the Sociometric Badges [49], putting them on at the time they entered the institution’s premises and taking them off only when leaving. Issues concerning device maintenance—for instance battery charging, data downloading, etc.—were taken care of by the study’s staff. An experience sampling methodology (ESM) [50] was employed to collect information about transient *personality states*. A similar procedure of experience sampling was adopted by [26,37]. Participants were asked to complete a short Internet-based survey three times a day (at 11.00, 14.00 and 17.00), automatically administered via email and they were granted a temporal window of 2.5 h to fill the survey before its expiration. We refer to the first survey as the morning survey, the second survey as the midday survey and the third survey as the afternoon survey. The survey requires the participants to describe their personality-related behaviours during the previous half hour. Hence, participants were asked to confirm their presence in the institute during the half hour before starting the questionnaire; only if confirmed, their responses would be included in the database.

### 2.3. Materials

#### 2.3.1. Personality states

**Figure 1.**
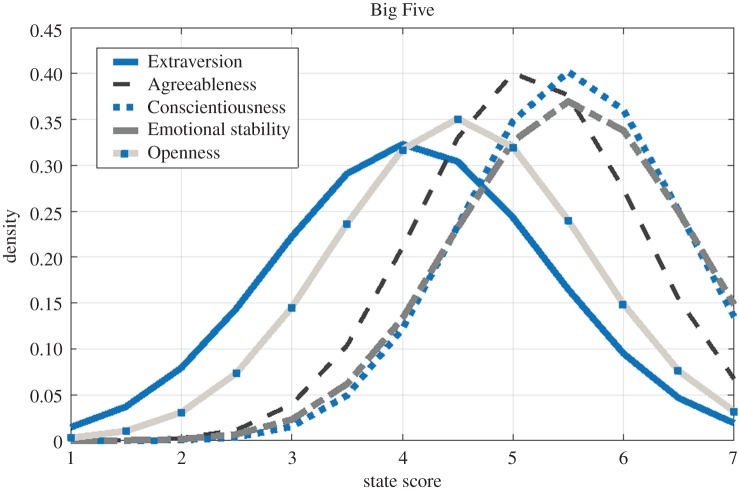
The average individual’s distributions of states over the entire study.

Personality states were assessed with the same format as traditional, adjective-based, Big Five scales with the exception that, rather than describing themselves in general, participants described their personality-related behaviours, feelings and thoughts during the previous half hour (e.g. ‘During the last half hour, how enthusiastic have you been?’). Specifically, the ten item personality inventory (TIPI) [51] was used to assess personality states. A 7-point scale ranging from 1=‘*Strongly* *Disagree*’ to 7=‘*Strongly* *Agree*’ was used for responses. Items were: ‘extraverted, enthusiastic’ and ‘reserved, quiet’ (reversed) for Extraversion; ‘calm, emotionally stable’ and ‘anxious, easily upset’ (reversed) for Emotional Stability; ‘sympathetic, warm’ and ‘critical, quarrelsome’ (reversed) for Agreeableness; ‘dependable, self-disciplined’ and ‘disorganized, careless’ (reversed) for Conscientiousness; ‘open to new experiences, complex’ and ‘conventional, uncreative’ (reversed) for Openness to Experience. The scores for each state were calculated by averaging the raw scores of the four corresponding items, with proper inversion when needed. Table 1 reports basic dataset statistics (median, mean, standard deviation, minimum and maximum values, skewness and kurtosis) on the participants’ personality states. Figure 1 shows the average individual’s distributions of states over the entire study.

**Table 1.** Descriptive statistics for personality states.

	Extraversion	Agreeableness	Conscientiousness	Emotional Stability	Openness
median	4	5.5	5.5	6	4.5
mean	4.07	5.13	5.53	5.54	4.51
s.d.	1.23	0.99	0.99	1.08	1.14
min.	1	1	1	1	1
max.	7	7	7	7	7
skewness	0.15	−0.86	−0.93	−1.09	0.02
kurtosis	2.76	4.15	3.93	4.01	2.80

#### 2.3.2. Sociometric Badges

The participants used wearable sensors called Sociometric Badges [49] every working day within the institution to record the social interactions between the participants in the study. These sensors are equipped with accelerometers, audio, Bluetooth and infrared sensors to, respectively, capture: body movements, prosodic speech features, co-location with other individuals and face-to-face interactions. Sociometric Badges have been used in several studies to capture face-to-face communication patterns, relationships among individuals, collective behaviour and performance outcomes, such as productivity [52] and job satisfaction [53].

In this paper, we use badges to track face-to-face interactions by means of infrared sensors that recognize similar sensors facing them, implying that the two participants wearing them had a conversation or eye contact. For a badge to be detected through infrared sensors from another badge, the two badges must have a direct line of sight and the receiving badge’s infrared must be within the transmitting badge’s infrared signal cone of height *h*≤1 *m* and a radius of *r*≤*h* tan *θ*, where *θ*=15^°^; the infrared transmission rate (TRir) was set to 1 Hz. When the infrared sensor of a participant *X* detects the sensor of a participant *Y* , a face-to-face contact is detected between *X* and *Y* .

### 2.4. Data preprocessing

To use graphlets including information on the state of the alters, personality states scores were dichotomized using the median as the threshold value (Low<median and High≥median). Specifically, we use a sample-based threshold in order to effectively capture whether significant transitions between High and Low states in a person may influence the others they get in contact with. In this way, we avoid labelling as ‘High’ participant *X*’s states reporting lower scores than ‘Low’ states of participant *Y* ; this situation may instead happen using a person-based threshold. Hence, the level of each participant in each dynamic state at a given survey is identified as being one of the two levels, either Low or High. State dynamics consist of changes (or lack thereof) of levels between two consecutive surveys for each participant in a given day. The possible transitions are Low to Low (L→L), Low to High (L→H), High to Low (H→L) and High to High (H→H). The extracted transitions and their distribution among the dataset are given in table 2.

**Table 2.** Number of transitions between Low/High (L/H) levels for personality states.

Transition	Extraversion	Agreeableness	Conscientiousness	Emotional Stability	Openness
L → L	311	264	290	299	325
L → H	392	300	322	270	348
H → L	422	328	373	335	380
H → H	753	986	893	974	825

For each of the four types of transitions as described above, we extracted the number of face-to-face interactions with other participants (*alters*) detected between two consecutive surveys. We used the lagged levels of the personality states of the other participants (*alters*) the individual is interacting with: for a transition taking place between the morning survey (time *t*) and the midday survey (time *t*+1), we consider the personality states’ scores of *alters* recorded up to the morning survey (time *t*).

The data comprise 3220 surveys by the 54 participants. The response rate for the surveys was 83.9% [54]. Ideally, the number of filled surveys should be 4860 (54 participants×3 daily surveys×30 working days); however, participants reported absences from work for a variety of reasons, such as attending conferences, being absent due to illness, working from home and leaving work early, reducing the number of expected responses. Additionally, we only addressed transitions in states levels between morning and midday surveys and transitions in states levels between midday and afternoon surveys. Therefore, the number of daily expected transitions is 2. Missing surveys have an impact on the number of transitions: if a participant did not fill in the midday daily survey, then two transitions will be absent for that participant.

The infrared interaction data are expected to be symmetric, as one badge can only register a face-to-face contact if it is in the direct line of sight of the other badge; however, we found some asymmetric interactions (e.g. the participant *X*’s infrared detected the participant *Y* ’s infrared, while the participant *Y* ’s infrared did not). We took a corrective measure by adding the missing data when required.

### 2.5. Graphlets

We encode the face-to-face interactions between participants as *graphlets*, defined as induced subgraphs of a larger network. In Bioinformatics and Computational Biology domains, graphlets have been introduced for the study of large biological networks, for example, network alignment [55]. Recently, graphlet analysis has been also applied to Facebook messaging and historical crime data [56]. Here, we investigate their effectiveness in the context of human interaction networks.

**Figure 2.**
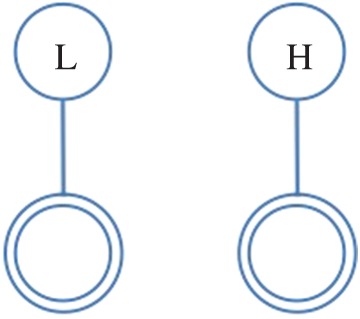
Graphlet configurations used. Bottom nodes (double circled) represent the reference participant (the ego), while top nodes represent alters and their binary states. One-to-one interaction with state level Low and High in unit time (15 min) time window. Participant interacts with two alters in unit time window. H, High; L, Low.

**Figure 3.**
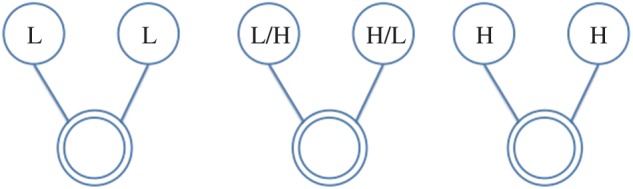
Participant interacts with two alters in unit time window. H, High; L, Low.

**Figure 4.**
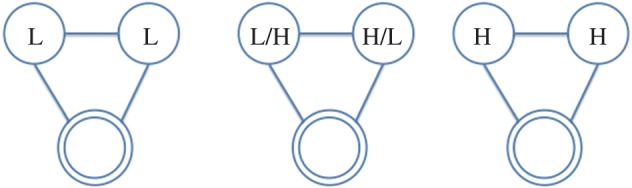
Participant interacts with two alters, who are interacting with each other. H, High; L, Low.

**Figure 5.**
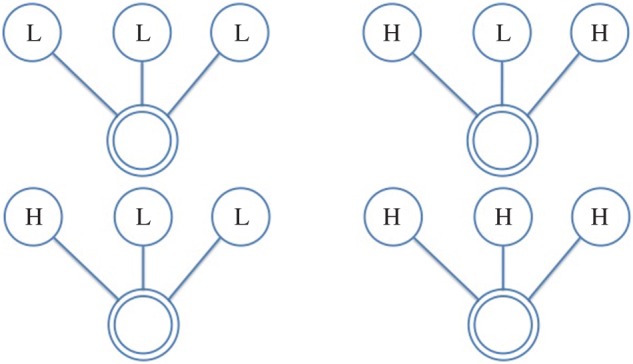
Participant interacts with three alters in unit time, who are not interacting with each other. H, High; L, Low.

**Figure 6.**
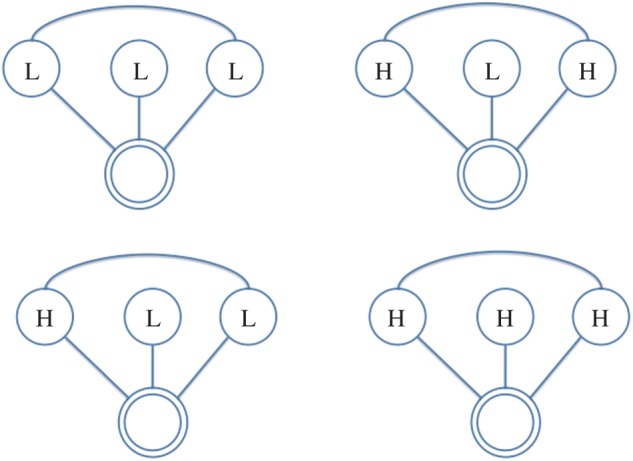
Participant interacts with three alters in a unit time, where two alters are interacting with the participant and each other, while the remaining alter is only interacting with the participant. H, High; L, Low.

**Figure 7.**
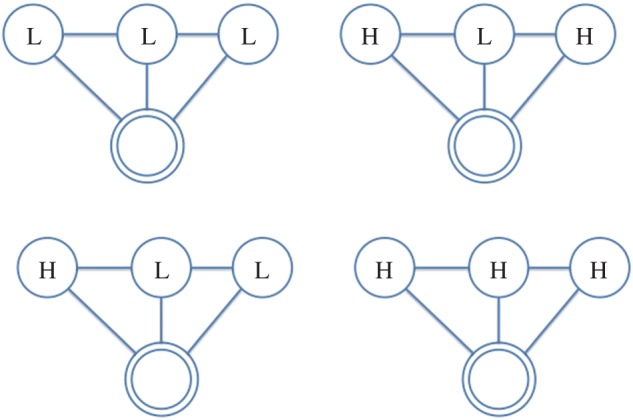
Participant interacts with three alters in unit time, where all the three alters are interacting with two other alters. H, High; L, Low.

**Figure 8.**
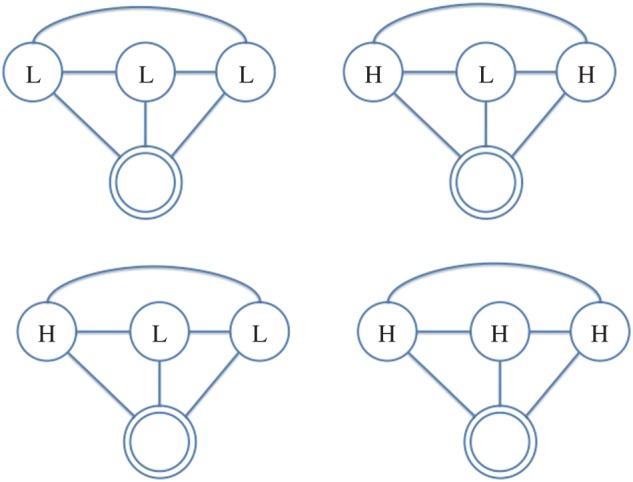
Participant interacts with three alters in unit time, who are all interacting with each other. H, High; L, Low.

In this paper, we use the following terminology: all individuals are represented as *nodes* in a network, with interactions between them acting as the *ties* which connect them; further, we refer to the individual under consideration as the *ego*, and the nodes they are connected to as their *alters*. Hence, the *ego-network* is a partial view on the global interaction network, centred on a given individual (the *ego*), including only those nodes (the *alters*) connected to them and the ties (if present) between them. From the network of face-to-face interactions between our participants, we extract the graphlets for each of them (i.e. specific network configurations observed in their *ego-networks*), which represent their local interactions. We consider all possible graphlets up to four nodes, as shown in figures 2–8, where the double circle represents the ego, and their alters and the possible multiple patterns of reciprocal interactions. The graphlets embed information on the current (binary) state of the alters, but not of the ego, in order to account for possible social influence effects. Further, we use the terms *dyads*, *triads* and *tetrads* to encode the ego’s interaction with one, two or three alters, respectively.

More specifically, we extract graphlets from infrared sensor data starting from 30 min before the first survey and until 30 min before the second survey was filled out. We discretize each time window into 15 min slices in order to represent the evolution of the interaction patterns over time, taking into consideration the alters’ states in order to account for the situational influence effects. To keep our model simple, the graphlets are not weighted; in other words, we do not account for multiple face-to-face interactions with the same individual over the same 15 min slice. To build a histogram, we thus count the occurrences of graphlet configurations in 15 min slices, and then proceed to sum the histograms obtained for each time slice and obtain a feature vector representative of the window under analysis. Hence, interactions in a 15 min slice will be encoded by a histogram with as many columns as the possible graphlet configurations; accordingly, the final histogram resulting from their sum over the 3 h period will have the same dimensions. Finally, we use these vectors as the independent variables in our regression models. In table 3, we report the possible labelling configurations for graphlets in our analyses while the graphlet representations are portrayed in figures 2–8; as we also account for all possible interaction configurations, our independent variables (each reflecting a labelling configuration according to table 3) consist of:

— dyads, representing the *ego* interacting with one *alter*;
— triads, representing the *ego* interacting with two *alters*, further distinguishing between *open* (i.e. no tie/interaction between the alters) and *closed* (i.e. there is a tie/interaction between the alters);
— tetrads, representing the *ego* interacting with three *alters*, which can occur in further four configurations, depending on how many alters interact, and hence have a tie, between each other.

**Table 3.** Basic graphlet configurations labelled by personality states. H, High; L, Low.

Dyad	Triad	Tetrad
L	LL	LLL
H	HL	HLH
	HH	HLL
		HHH

To sum up, the list of independent variables in our study thus comprises: *Dyad*_***state***, *Triad*_***type***_***state***, *Tetrad*_***type***_***state***, where **state** encodes the personality state labels as in table 3, while **type** takes a value between (*Open, Closed*) for triads, and (*NoAlterTie, OneAlterTie, TwoAlterTies, ThreeAlterTies*) for tetrads.

The extraction of graphlets from infrared data was performed by using Matlab, a numerical programming language. The code is accessible through Github.

### 2.6. Statistical models

For each possible transition between the two (High/Low) levels of a particular state (e.g. from High Extraversion to Low Extraversion), we fit a distinct logistic regression model where the binary dependent variable (DV) denotes the presence or absence of a transition (0=‘no transition’ and 1=‘transition’). The independent variables (IVs) are the number of graphlet configurations computed over the transition window (e.g. from the morning survey to the midday survey). In each distinct logistic regression model, all the IVs are entered at the same time as a block. Let *X*→*Y* denote a transition by the ego from level *X* to level *Y* of a state *S* (*X*=*Y* denotes stability). Let *p*(*X*→*Y*) be the probability of this transition between two consecutive surveys. For each dynamic state *S*, we fit the following model:

2.1 $$\ln\left(\frac{p(X\to Y)}{1-p(X\to Y)}\right)=\alpha +\beta_{X},$$

where *α* is a constant (intercept) and *β*_*X*_ are coefficients of the *main* effects of the independent variables. This model allows us to probe the effects that contingent aspects (different structural configurations of interactions with various types of alters) have on the probability of a transition (i.e. High to Low, Low to High or lack thereof) within a given state. The correlation between each IV and the DVs are captured by means of standardized *β* coefficients. The interpretation of the standardized *β* coefficient is the following:

— when the value of standardized *β* is 0 for a given IV, there is no association between the IV and the DV;
— when the value of standardized *β* is greater than 0, there is a positive relationship between the IV and the DV. In this case, a one unit increase in the IV (e.g. number of single interactions with alters in level L, number of dyadic interactions with alters both in level L) corresponds to an *increase* in the probability of a transition between states; and
— when the value of standardized *β* is less than 0, there is an inverse relationship between the IV and the DV such that a one-unit increase in the IV corresponds to a *decrease* in the probability of a transition between states.

As our data consist of multiple responses from each individual over the course of the study, we would expect to have correlations within and between observations of participants. Multiple responses from the same individual cannot be assumed to be independent from one another and hence the use of linear models violates the independence assumption, as the responses are inter-dependent rather than independent. To deal with this inter-dependence, we used linear mixed models adding a random effect for each participant. Individual differences can be modelled by assuming different random intercepts for each individual. These random effects characterize the idiosyncratic variation that is due to individual differences [57].

The linear mixed model, used in this analysis, is an extension of the general linear model, in which factors and covariates are assumed to have a linear relationship to the dependent variable. Fixed-effects factors are thought of as variables whose values of interest are represented in the data file, while random effects are variables whose values can be considered a random sample from a larger population of values. The random effects can be useful for explaining excess variability in the dependent variable [58].

Regression results are described in the following section, discussing only coefficients with a significance level of *p*<0.05.

The analyses with linear mixed models were performed by using R, a statistical programming language. The code is accessible through Github.^1^

## 3. Results

The presentation of the results is divided in three parts. First, the distribution of *personality states* across time within the average individual is described. Specifically, the amount of *within-person* variability is computed and then compared with the amount of *between-person* variability. Second, the role played by different structural configurations of face-to-face interactions in predicting the variability of *personality states* is tested. Finally, the *goodness of fit* for each of the models examined is shown.

### 3.1. Evidence for personality states approach

Following [26], we computed the *within-person* variance and the *between-person* variance. The *within-person* variance is the average of 54 *within-person* variances; representing how much the average of the individual’s personality states differ over time. Specifically, the *within-person* variance is assessed by first calculating five variances per participant, one for each personality trait (e.g. Extraversion, Emotional Stability, etc.), with each variance representing the amount that the individual varies in how they manifest a given trait. Then, the averages, across individuals, of these variances are computed. The *between-person* variance is assessed by calculating the variance across personality state mean levels from the experience sampling, representing how much the individuals differ from each other in their average levels.

Interestingly, the *within-person* variance tends to be higher than the *between-person* variance, a trend that is stronger for Extraversion than for the other states (table 4). Thus, individuals tend to differ more from themselves over time than from each other at the average level.

**Table 4.** Variability of personality states. *Within-person* variance is the average of within-person variances; while *between-person* variance is the variance across personality state mean levels from the experience sampling.

				Emotional	
	Extraversion	Agreeableness	Conscientiousness	Stability	Openness
within-person	1.14	0.63	0.57	0.69	0.80
between-person	0.41	0.38	0.44	0.49	0.52

In addition, we computed the intraclass correlation (ICC1) in order to capture the proportion of total variance accounted for by the *within-person* variation. The theoretical formula for ICC1 is as follows:

3.1 $$\mathrm{ICC}1=\frac{\rho^{2}(b)}{\rho^{2}(b)+\rho^{2}(w)},$$

where *ρ*^2^(*b*) stands for *between-person* variance, and *ρ*^2^(*w*) stands for *within-person* variance. In particular, an ICC1 score close to 1 indicates high similarity between values from the same group, while an ICC1 score close to zero means that values from the same group are not similar. Our results show that the proportion of total variance accounted for by the *within-person* variation is 0.27 for Extraversion, 0.38 for Agreeableness, 0.44 for Conscientiousness, 0.42 for Emotional Stability and 0.40 for Openness.

In sum, the obtained results are in line with findings in the literature on personality states [26] and emphasize the importance of shifting the attention to *within-person* variations and their dependence on situational factors when addressing the interplay between personality and actual behavioural manifestations.

### 3.2. The association between social interactions and personality states dynamics

In a preliminary analysis stage, we found no occurrences for 14 of 20 graphlet configurations. Specifically, we have no occurrences of tetrad graphlets where the alters were interacting with each other. This might be due to the complex nature of the interactions and the relatively short time-frame that the features were calculated for. Hence, the configurations used in the study were: the dyadic interactions, the open and closed triadic interactions and the tetrad interactions with no connections for the multiple alters. The results are reported here divided by personality state for each of the four types of transitions of personality state levels of Low and High. Significant findings are marked with an asterisk; * denotes significance at the level of *p*<0.05, ^**^ at the level of *p*<0.001 and finally ^***^ at the level of *p*<0.0001. It is worth noting that some large standardized *β* coefficients are not significant. For example, 0.232, −0.206, −0.326 and 0.771 when the participants experience closed triadic interactions with alters who are both in High states (table 5). An explanation might be that the dispersion (standard deviation, standard error) is very high and hence it lowers the significance. Relatedly, it might be a problem of *statistical power* due to the limited size of the sample. Indeed, we have very few examples of closed triadic interactions with alters who are both in High states.

**Table 5.** Linear mixed models results—Extraversion. The values reported are standardized *β* coefficients. H, High; L, Low.

	L → L	L → H	H → L	H → H
intercept	0.193^***^	0.233^***^	0.212^***^	0.361^***^
Dyad_L	−0.019*	−0.024*	0.000	0.042^***^
Dyad_H	−0.022^***^	−0.011	0.008	0.024^**^
Triad_Open_HL	0.008	0.048	0.028	−0.090^**^
Triad_Open_LL	−0.054	0.022	0.052	−0.019
Triad_Open_HH	−0.005	−0.061*	0.019	0.048
Triad_Closed_HH	−0.232	−0.206	0.7719	−0.326
Tetrad_NoAlterTie_HLH	0.061	−0.094	0.003	0.052
Tetrad_NoAlterTie_HLL	−0.036	−0.057	−0.043	0.039
Tetrad_NoAlterTie_LLL	0.092	0.029	0.013	−0.136
Tetrad_NoAlterTie_HHH	0.088	−0.065	−0.090	0.069

#### 3.2.1. Extraversion

The results for Extraversion are reported in table 5. The probability to move from Low to High is negatively associated with dyadic interactions with alters in Low state (*β* −0.024, 95% CI [−0.04,−0.00]) and with triadic interactions with alters who are both in High states (*β* −0.061, 95% CI [−0.12,−0.01]). Hence, we may argue that Low alters have an *inertial* effect on individuals in the same level of the state. Indeed, those who are in the Low level of the state tend not to move to a higher level when they interact with alters in the Low level of state, in dyadic interactions. A similar finding was recently reported in [42]. Instead, the interaction with two alters who are High in Extraversion makes it less likely from an ego to move from a Low state to a High state, an example of *repulsion*.

Furthermore, staying at a Low state of Extraversion is negatively associated with dyadic interactions with alters who are in Low (*β* −0.019, 95% CI [−0.04,−0.00]) or in High (*β* −0.022, 95% CI [−0.03,−0.01]) states. On the contrary, an ego is more likely to remain in a High state when interacting with alters who are in Low (*β* 0.042, 95% CI [0.02, 0.07]) or in High states (*β* 0.024, 95% CI [0.01, 0.04]). Thus, our results show that interacting with one alter (regardless of their state) reduces the likelihood of a Low ego staying Low and increases the probability of a High ego staying High. Instead, the probability of an ego to remain in a High state decreases with triadic interactions with High and Low alters (*β* −0.090, 95% CI [−0.15,−0.03]), hence these interactions have a *push* effect on the ego.

Finally, we found no significant effects for the transition from High to Low.

#### 3.2.2. Agreeableness

 Table 6 reports the results for Agreeableness. The results show that a transition from a Low to High state is more likely when the participants have been experiencing triadic interactions with alters who are both in a High state (*β* 0.053, 95% CI [0.01, 0.10]). This is evidence of an *adaptive* behaviour, namely *attraction*. As defined in the introduction, *attraction* happens whenever increased interaction with alters in a state level different from the ego’s corresponds to either an increased probability for the ego to move towards that level or a decreased probability to move away from that level.

**Table 6.** Linear mixed models results—Agreeableness. The values reported are standardized *β* coefficients. H, High; L, Low.

	L → L	L → H	H → L	H → H
intercept	0.146^***^	0.168^***^	0.180^***^	0.500^***^
Dyad_L	−0.005	−0.013	0.001	0.020
Dyad_H	−0.002	−0.005	−0.003	0.010
Triad_Open_HL	0.008	−0.031	0.021	0.002
Triad_Open_LL	0.121*	−0.057	0.015	−0.078
Triad_Open_HH	−0.012	0.053*	−0.021	−0.018
Triad_Closed_HL	−0.091	−0.118	−0.160	0.270
Tetrad_NoAlterTie_HLH	−0.081	−0.015	−0.023	0.122*
Tetrad_NoAlterTie_HLL	0.001	0.029	−0.109	0.047
Tetrad_NoAlterTie_LLL	0.203	0.182	−0.031	−0.346
Tetrad_NoAlterTie_HHH	0.035	0.036	−0.069	−0.003

The stability of the ego in a Low state is positively associated with triadic interactions with alters who are both in a Low state (*β* 0.121, 95% CI [0.03, 0.22]). Instead, when the ego remains in a High state, there is a positive relationship with complex interactions with three alters, two in a High state and one in a Low state, without any connection to each other (*β* 0.122, 95% CI [0.00, 0.24]).

Similar to Extraversion, we did not find significant effects for the transition from High to Low in Agreeableness.

#### 3.2.3. Conscientiousness

The results for Conscientiousness are reported in table 7. The results show that a transition from Low to High is positively associated (*β* 0.120, 95% CI [0.01, 0.23]) with complex interactions where three alters, two of whom are in a High state and one in a Low state, are not interacting with each other. Transitions from High to Low are negatively associated with dyadic interactions with High alters (*β* −0.015, 95% CI [−0.03,−0.00]). Hence, alters seem to have an *inertial* effect on the ego transition for those egos who are high in Conscientiousness.

**Table 7.** Linear mixed models results—Conscientiousness. The values reported are standardized *β* coefficients. H, High; L, Low.

	L → L	L → H	H → L	H → H
intercept	0.148^***^	0.1725^***^	0.2203^***^	0.4512^***^
Dyad_L	0.004	0.002	0.000	0.000
Dyad_H	0.000	−0.003	−0.015*	0.022*
Triad_Open_HL	0.091^***^	0.002	−0.050	−0.035
Triad_Open_LL	−0.089	−0.066	−0.057	0.213^***^
Triad_Open_HH	−0.005	−0.007	−0.021	0.038
Triad_Closed_HH	−0.080	−0.178	−0.194	0.455
Tetrad_NoAlterTie_HLH	−0.048	0.120*	0.041	−0.117
Tetrad_NoAlterTie_HLL	0.042	−0.006	0.122	−0.138
Tetrad_NoAlterTie_LLL	−0.267	−0.180	0.213	0.270
Tetrad_NoAlterTie_HHH	0.045	0.036	−0.073	−0.020

Remaining in a Low state is associated with triadic interactions with alters who are in mixed states, High and Low (*β* 0.091, 95% CI [0.04, 0.14]). Finally, when the participant remains in a High state, there is a positive relationship with dyadic interactions with High alters (*β* 0.022, 95% CI [0.00, 0.04]) and with triadic interactions with alters who are both in a Low state (*β* 0.213, 95% CI [0.09, 0.34]).

#### 3.2.4. Emotional Stability

The results for Emotional Stability can be found in table 8. The transition from Low to High is positively associated with complex interactions where the ego is interacting with three alters who are not connected to each other and the majority of alters are in the same state the ego is transitioning to (*β* 0.124, 95% CI [0.02, 0.23]). Hence, this complex interaction configuration seems to *attract* the ego.

**Table 8.** Linear mixed models results—Emotional Stability. The values reported are standardized *β* coefficients. H, High; L, Low.

	L → L	L → H	H → L	H → H
intercept	0.162^***^	0.145^***^	0.172^***^	0.5223^***^
Dyad_L	0.012	0.015	−0.006	−0.021
Dyad_H	−0.003	−0.011	0.010	0.002
Triad_Open_HL	0.016	−0.015	0.038	−0.047
Triad_Open_LL	0.017	0.076	−0.023	−0.072
Triad_Open_HH	−0.033	0.014	0.007	0.012
Triad_Closed_HH	−0.049	−0.144	−0.091	0.159
Tetrad_NoAlterTie_HLH	−0.052	0.124*	−0.097	0.007
Tetrad_NoAlterTie_HLL	0.016	−0.100	0.023	0.059
Tetrad_NoAlterTie_LLL	−0.156	−0.043	0.194	−0.032
Tetrad_NoAlterTie_HHH	0.003	−0.052	−0.054	0.104

#### 3.2.5. Openness to experience

The results for Openness to Experience can be found in table 9. For this personality dimension, a transition from Low to High is positively correlated with a complex interaction where three Low alters have no connection to each other (*β* 0.277, 95% CI [0.02, 0.54]). Interestingly, Low alters seem to *push* the ego to a higher level. When the participant remains in a High state, there is a significant positive relationship of dyadic interactions with alters of a High state (*β* 0.022, 95% CI [0.00, 0.04]). From this we can infer that remaining in a High state is co-occurring with interactions with others who are in the same state.

**Table 9.** Linear mixed models results—Openness to Experience. The values reported are standardized *β* coefficients. H, High; L, Low.

	L → L	L → H	H → L	H → H
intercept	0.172^***^	0.192^***^	0.207^***^	0.426^***^
Dyad_L	0.010	−0.010	0.012	−0.011
Dyad_H	−0.009	−0.010	0.003	0.022*
Triad_Open_HL	0.000	0.019	−0.019	0.001
Triad_Open_LL	0.052	−0.035	−0.084	0.061
Triad_Open_HH	0.032	0.022	−0.057	0.009
Triad_Closed_LL	−0.218	−0.177	−0.121	0.537
Tetrad_NoAlterTie_HLH	0.032	0.045	−0.030	−0.047
Tetrad_NoAlterTie_HLL	0.059	0.036	−0.109	0.014
Tetrad_NoAlterTie_LLL	0.062	0.277*	−0.130	−0.211
Tetrad_NoAlterTie_HHH	−0.064	0.054	−0.082	0.085

### 3.3. Goodness of fit

 Table 10 shows the goodness of fit for each of the models examined in our study. The goodness of fit is calculated as the variance (*R*^2^) explained by the models. There are two types of *variance explained* *R*^2^, (i) *marginal* *R*^2^ and (ii) *conditional* *R*^2^ as detailed in [59]. Marginal *R*^2^ shows the variance explained by fixed effects, while conditional *R*^2^ shows the variance explained by fixed and random effects. In our case, the marginal *R*^2^ is more relevant to quantify the absolute goodness of model fit as it calculates the variance explained by the fixed effects which comprise the graphlets’ features.

**Table 10.** Goodness of fit—marginal and conditional *R*^2^ values for graphlet-based models.

	marginal *R*^2^	conditional *R*^2^
	Extraversion	
L → L	0.011	0.029
L → H	0.012	0.028
H → L	0.005	0.006
H → H	0.017	0.069
	Agreeableness	
L → L	0.008	0.051
L → H	0.007	0.028
H → L	0.003	0.014
H → H	0.007	0.124
	Conscientiousness	
L → L	0.010	0.059
L → H	0.004	0.013
H → L	0.009	0.016
H → H	0.013	0.123
	Emotional Stability	
L → L	0.004	0.069
L → H	0.011	0.016
H → L	0.005	0.032
H → H	0.006	0.123
	Openness to Experience	
L → L	0.005	0.025
L → H	0.005	0.013
H → L	0.008	0.010
H → H	0.007	0.052

We also ran a series of ANOVA comparisons to measure whether the null models, which include only the intercept, are significantly different from the graphlet-based models, which comprise the graphlets’ features. While we found that the variance accounted for by the graphlet-based models is always greater than the one by the null models, this difference in variance is not always significant, as shown in table 11. In particular, for Agreeableness and Openness to Experience, for which we found few significant standardized *β* coefficients, the difference is not significant for any of the transitions investigated.

**Table 11.** Conditional *R*^2^—ANOVA comparisons between null and graphlet-based models. Significance at the level of *p*<0.05 is marked as *, while significance at the level of *p*<0.001 is marked by ^**^.

	conditional *R*^2^		
	_______________________________		
	null	graphlet	ANOVA
	Extraversion		
L → L	0.021	0.029	20.36*
L → H	0.016	0.028	21.97*
H → L	0.001	0.006	9.75
H → H	0.055	0.069	29.76^**^
	Agreeableness		
L → L	0.044	0.051	15.57
L → H	0.019	0.028	14.02
H → L	0.012	0.014	5.20
H → H	0.117	0.124	14.03
	Conscientiousness		
L → L	0.051	0.059	19.84*
L → H	0.008	0.013	8.34
H → L	0.007	0.016	15.87
H → H	0.106	0.123	26.83*
	Emotional Stability		
L → L	0.066	0.069	8.40
L → H	0.004	0.016	21.40*
H → L	0.026	0.032	10.31
H → H	0.117	0.123	12.84
	Openness to Experience		
L → L	0.020	0.025	8.75
L → H	0.007	0.013	10.10
H → L	0.003	0.010	15.10
H → H	0.041	0.052	13.78

## 4. Discussion

The purpose of this study was to investigate the role played (if any) by specific situational factors, that is, face-to-face interactions, in the manifestation of trait contents in *personality states*. In particular, we tested whether different structural characteristics of the face-to-face interaction network are predictors of the dynamic personality states of a given individual (the ego). To this end, we represented interactions between people as *graphlets*, induced subgraphs representing specific patterns of interactions, and designed regression analyses with the target of predicting dynamic transitions of participants’ self-reported personality states.

The obtained results show that within-person variability in the Big Five personality is indeed associated with variation in face-to-face interactions (*Hypothesis 1*). In line with the recent findings of [42], we also show that personality states are not caught from someone else; they are not the result of mere contact, but from the ways individuals (possibly unconsciously) respond to other people’s behaviour by either adapting to it—as discussed by social psychologists under the rubric of the ‘perception behaviour link’ or ‘chameleon effect’ [44]—or diverging from it. Again in line with the findings of [42], our results reveal more nuanced effects like *attraction*, *inertia*, *push* and *repulsion* (*Hypothesis 2*). As explained in the Introduction, both *attraction* and *inertia* represent a tendency of egos to adapt to their alters, whereas *push* and *repulsion* represent the tendency to complement the behaviour of others. We can, therefore, subsume these effects under the adaptation versus complementarity distinction [44].

Interestingly, the effects differ by type and level of the personality states (*Hypothesis 3*): we find evidence of adaptive behaviours for Agreeableness and Emotional Stability; while complementarity behaviours seem to characterize Openness to Experience. For example, our results indicate that people are more likely to move from Low to High Agreeableness when they interact with two alters who are High in Agreeableness, which is a case of *attraction*, a form of *adaptive behaviour*. Similarly, people are more likely to make a transition from Low to High Emotional Stability when they interact with three alters who are not connected to each other and the majority of alters are in the same state the ego is transitioning to. Thus, this complex configuration is another case of *attraction*. Instead, Low alters in Openness to Experience seem to *push* the ego to a higher level, a form of *complementarity*. Table 12 summarizes how the results obtained for each personality type fit with the different effects: *attraction*, *inertia*, *push* and *repulsion*.

**Table 12.** Summary table showing how the results for each trait fit with the different effects (e.g. *attraction, inertia, push, repulsion*). In the table, Extraversion is abbreviated as Extr., Agreeableness as Agree., Conscientiousness as Consc., Emotional Stability as Emo.Sta.

		Extr.	Agree.	Consc.	Emo. Sta.	Openness
adaptation	attraction				+	
	inertia	+	+			
complementarity	push	+				+
	repulsion	+				

Hence, one might argue that for Agreeableness and Emotional Stability the same level of personality states of all group members make the face-to-face interaction more stimulating; while if all group members have the same level of Openness to Experience, the face-to-face interaction might be less stimulating [60]. Additionally, VanderZee *et al.* [61] and Gibbons & Buunk [62] found that individuals low in Emotional Stability display a higher need for comparison. Moreover, they found that association between adaptation behaviours and Low states in Emotional Stability is consistent with the fact that a primary component for a low Emotional Stability (i.e. neuroticism) is represented by uncertainty about the self. However, further studies are required to explain these associations and to investigate possible causal relationships.

The results of our study also show that dyadic, triadic and in a few cases tetradic configurations of social face-to-face interactions contribute to predict the dynamic transitions of personality states. More complex interactions have very little impact on the transition of a participant’s personality states. Indeed, we have no occurrences of tetrad graphlets where the alters were interacting with each other. This might be due to the complex nature of the interactions and the relatively short time-frame that the features were calculated for.

Overall, our findings provide additional evidence that within-person variability in personality is meaningful and is related to specific situational factors, that is, different configurations of face-to-face interactions, thus encouraging further research to characterize face-to-face and social interactions in terms of their relevance to personality states [26,37,40,63,64].

Recent studies on personality and performance at work have started moving towards an integrative approach to personality that recognizes the relevance of both personality traits and personality states [65–68]. For example, Judge *et al.* [67] argue that both traits and states are important for performance as illustrated by the fact that we not only expect a surgeon to be calm on average (High score in Emotional Stability trait), but also that they remain calm in both emergency and routine situations (High score in Emotional Stability state). Hence, the capability of modelling a possible transition of a given individual from a personality state *X* to a personality state *Y* , looking at their face-to-face interactions, make it possible to predict related outcomes such as performance at work.

Another important implication of our study is its relevance for planning behavioural change strategies and their possible impact on fields such as organizational behaviour and public health. Indeed, it becomes possible to design managerial, policy or clinical strategies to modify the personality states of one person, which can also have a social influence effect on others, thereby enhancing the efficacy and cost effectiveness of the intervention. Moreover, some recent studies have shown that extraverts experience more positive affect than introverts because they actually enact more extraverted states in their daily lives [69,70]. In contrast to trait-based explanations, which treat the relationship between Extraversion and positive affect as immutable, this dynamic explanation raises the possibility that introverts, who already act extraverted on some occasions [26] and have the ability to act extraverted on demand [69,71], might be able to increase their happiness levels by enacting more extraverted states in their daily lives. Hence, we may use face-to-face interactions not only to modify the personality states of one person but also related outcomes such as happiness levels.

From a methodological perspective, our study demonstrates that the automatic sensing of social interactions can build a detailed picture of the dynamics of personality states in a work organization. In our approach, we only use the number of infrared contacts to measure the structural configurations of face-to-face interactions and to predict the ego’s transitions in personality states. Neither self-reported relationships nor content of interactions were used to investigate the impact of alters on one’s states: such drastic design choice allows for minimizing the participants’ biases and fully preserving their right to privacy and confidentiality. Given the pervasiveness of the infrared sensor and its finer grained perspective compared with methodologies that exclusively rely on surveys, we may argue that Sociometric Badges and similar wearable devices [47,49,72] should be seriously considered as new research tools for personality psychology [73].

We conclude by acknowledging some limitations of this study, starting from the relatively small size of the sample (54 participants) and the variance lost dichotomizing the personality states, a necessary strategy in order to use graphlets in our analyses. Other limitations are the non-availability of behavioural data and self-reported personality states during the hours spent outside of work (e.g. evenings at home, weekends), the non-availability of behavioural data concerning the interaction with people not participating in the study, and, as previously mentioned, the non-availability of content of communication interactions. As a consequence, we may ignore in our analyses face-to-face interactions relevant in causing changes in personality states. Moreover, our study identifies only correlations and cannot support causal conclusions about face-to-face interactions and personality states. Recently, [74] identified situations in which latent *homophily* (similarity among interaction partners) cannot be distinguished, observationally, from *contagion* (the adaptation of behavioural responses to the behaviour of other people). Accordingly, they argue that this raises barriers to many inferences social psychologists and sociologists would like to make about the underlying causal mechanisms. Hence, just as *contagion* and *homophily* may be confounded in observational data [74], it may be the case that our *complementarity* effects may be confounded with a corresponding phenomena like *heterophily* (the tendency to interact preferentially with people who exhibit opposing behaviour or traits). However, the theoretical approach [37,63,64] assumes that situational characteristics cause changes in behaviour as individuals adapt to the situation, so it is important to eventually establish causality. A possible solution would be conducting focused experimental studies to test the causal direction of the associations between face-to-face interactions and personality states. For example, some configurations of face-to-face interactions could be manipulated to test whether it causes a change in personality states. Finally, our study does not investigate the influence of individuals on situations in terms of selecting or modifying situations [75].

Regardless of these limitations, our study provides a first step towards the investigation of different structural characteristics of face-to-face interactions as predictors of the variability in personality states.

## Footnotes

1 See https://github.com/didemgundogdu/RoyalOpenSciencePersonalityDynamics.

## Data accessibility

Datasets and codes, which are used in this study are available via the GitHub Repository (https://github.com/didemgundogdu/RoyalOpenSciencePersonalityDynamics).
